# Prognostic Value of Coronary Artery Calcification in Patients with COVID-19 and Interstitial Pneumonia: A Case-Control Study

**DOI:** 10.3390/jcdd11100319

**Published:** 2024-10-11

**Authors:** Gianni Dall’Ara, Sara Piciucchi, Roberto Carletti, Antonio Vizzuso, Elisa Gardini, Maria De Vita, Chiara Dallaserra, Federica Campacci, Giovanna Di Giannuario, Daniele Grosseto, Giovanni Rinaldi, Sabine Vecchio, Federica Mantero, Lorenzo Mellini, Alessandra Albini, Emanuela Giampalma, Venerino Poletti, Marcello Galvani

**Affiliations:** 1 Cardiology Unit, Morgagni-Pierantoni Hospital, 47121 Forlì, Italy; 2 Department of Medical and Surgical Sciences (DIMEC), University of Bologna, 47121 Forlì, Italy; 3 Department of Radiology, Morgagni-Pierantoni Hospital, 47121 Forlì, Italy; 4 IRCCS Istituto Romagnolo per lo Studio dei Tumori “Dino Amadori”—IRST, 47014 Forlì, Italy; 5 Cardiology Unit, Infermi Hospital, 47923 Rimini, Italy; 6 Cardiology Unit, Ceccarini Hospital, 47838 Riccione, Italy; 7 Department of Radiology, Infermi Hospital, 47923 Rimini, Italy; 8 Cardiology Unit, Santa Maria delle Croci Hospital, 48121 Ravenna, Italy; 9 Department of Radiology, Santa Maria delle Croci Hospital, 48121 Ravenna, Italy; 10 Cardiology Unit, Bufalini Hospital, 47521 Cesena, Italy; 11 Department of Medical Specialties-Pneumology, Morgagni-Pierantoni Hospital, 47121 Forlì, Italy; 12 Department of Respiratory Diseases and Allergy, Aarhus University, 8000 Aarhus, Denmark; 13 Cardiovascular Research Unit, Myriam Zito Sacco Heart Foundation, 47121 Forlì, Italy

**Keywords:** coronary artery calcium, calcium score, coronavirus disease-19, COVID-19, severe acute respiratory syndrome coronavirus-2, SARS-CoV-2, pulmonary severity score

## Abstract

**Background:** Patients suffering from coronavirus disease-19 (COVID-19)-related interstitial pneumonia have variable outcomes, and the risk factors for a more severe course have yet to be comprehensively identified. Cohort studies have suggested that coronary artery calcium (CAC), as estimated at chest computed tomography (CT) scan, correlated with patient outcomes. However, given that the prevalence of CAC is gender- and age-dependent, the influence of baseline confounders cannot be completely excluded. **Methods:** We designed a retrospective, multicenter case-control study including patients with COVID-19, with severe course cases selected based on death within 30 days or requiring invasive ventilation, whereas controls were age- and sex-matched patients surviving up to 30 days without invasive ventilation. The primary outcome was the analysis of moderate-to-severe CAC prevalence between cases and controls. **Results:** A total of 65 cases and 130 controls were included in the study. Cases had a significantly higher median pulmonary severity score at chest CT scan compared to controls (10 vs. 8, respectively; *p* = 0.0001), as well as a higher CAC score (5 vs. 2; *p* = 0.009). The prevalence of moderate-to-severe CAC in cases was significantly greater (41.5% vs. 23.8%; *p* = 0.013), a difference mainly driven by a higher prevalence in those who died within 30 days (*p* = 0.000), rather than those requiring invasive ventilation (*p* = 0.847). White blood cell count, moderate-to-severe CAC, the need for antibiotic therapy, and severe pneumonia at CT scan were independent primary endpoint predictors. **Conclusions:** This case-control study demonstrated that the CAC burden was higher in COVID-19 patients who did not survive 30 days or who required mechanical ventilation, and CAC played an independent prognostic role.

## 1. Introduction

The severe acute respiratory syndrome coronavirus-2 (SARS-CoV-2) pandemic put a strain on healthcare systems worldwide until May 2023, and the incidence of new cases continues to be closely monitored. In the early stages of the pandemic, particularly during the first wave, most severe cases were due to the related respiratory syndrome. However, the factors making patients susceptible to an unfortunate course have yet to be comprehensively identified. Baseline comorbidities played an important role, particularly obesity, hypertension, diabetes mellitus, cardiovascular and cerebrovascular diseases [1,2]. Moreover, various cardiac complications occurred during the acute phase [3,4], often indicated by the presence and extent of myocardial damage, as reflected by cardiac troponin elevation, which, in turn, may be caused by occult coronary artery disease [5]. In addition to being the gold standard for diagnosing infection-related interstitial pneumonia, high-resolution computed tomography (CT) of the chest may also provide important information on the presence of coronary heart disease, allowing for the analysis of coronary artery calcification (CAC) distribution and extent, even in non-cardio-synchronized acquisitions. The degree of CAC detected by CT correlates with the total volume of atherosclerotic disease measured by invasive investigations [6]. The Maastricht Intensive Care COVID study demonstrated a correlation between CAC and the extent of myocardial injury during critical illness, expressed by a higher plasmatic level of high-sensitivity troponin-T [7]. 

Therefore, estimating coronary calcium in coronavirus disease-2019 (COVID-19) patients could be useful in predicting an unfavorable outcome if the presence of occult coronary heart disease significantly contributes to this process [8,9,10,11]. Several cohort studies, mainly derived from single-center experiences, have reported somewhat conflicting evidence, suggesting that CAC burden has an independent prognostic value for short-term mortality [12,13,14,15]. Le Hir et al. found that moderate or heavy CAC was associated with a higher 6-month mortality [16]. However, the prevalence of CAC being gender-dependent and the amount of CAC a marker of the subject’s biological age, cohort studies do not allow such prognostic impact to be assessed. Indeed, multivariable adjustments cannot eliminate the potential of bias when baseline differences are too great. To partially overcome this limitation and reach a higher step on the evidence ladder regarding CAC influence on COVID-19 patient outcomes, we designed a retrospective multicenter case-control study, focusing on data from the first wave of the pandemic, a period characterized by greater severity and a higher incidence of adverse events. We sought to compare the difference in the prevalence of CAC between patients suffering from COVID-19-related interstitial pneumonia with a severe course and those who did not die or require mechanical ventilation, and to evaluate whether coronary artery calcium was an independent predictor of an adverse 30-day outcome.

## 2. Material and Methods

### 2.1. Patient Population

We conducted a retrospective multicenter analysis of 2126 consecutive patients from 1 March to 30 April 2020 admitted to the Emergency Departments of four hospitals located in Forlì, Cesena, Ravenna, and Rimini, within the Romagna Area (1,124,785 inhabitants living in 6380.6 km^2^) with a confirmed COVID-19 diagnosis. Infection was detected with high-throughput sequencing or real time reverse-transcriptase polymerase-chain-reaction assay of nasal and pharyngeal swab specimens, according to the interim guidance of the World Health Organization. The study was approved by the ethics committee of the Local Health Agency of Romagna (CE-ROM).

### 2.2. Inclusion and Exclusion Criteria

Patients were eligible for inclusion if they met all the following criteria:

- availability of medical history, symptoms at presentation, and physical examination;
- confirmed COVID-19 diagnosis;
- chest CT imaging for suspected interstitial pneumonia at hospital admission.

We excluded patients with previous coronary revascularization (both percutaneous and surgical), since the presence of coronary stents or grafts can impair CAC score calculation, as well as those with inadequate image quality as judged by the reading radiologist.

### 2.3. Patient Selection

The study was a matched case-control study with two controls for each case. Cases were consecutive patients selected based on death occurring within 30 days from admission or requiring invasive ventilation during the index hospitalization. Controls were age- and sex-matched patients surviving up to 30 days without the need for invasive ventilation. We divided cases into age strata of 10 years, assuring within each age stratum twice the number of controls with an identical gender proportion. Each control was matched to a single case.

### 2.4. Chest CT Scan Scoring and Definitions

All the examinations were performed on at least 16-slice CT scanners (GE LightSpeed 16 Slice, Milwaukee, WI, USA). Acquisition was not ECG-gated, without contrast dye, with the patient in supine position, and obtained with a standard dose scanning protocol, reconstructed at 1.25 mm slice thickness, and 1.25 mm collimation. Lung window was set at −600 Hounsfield units (HU), and window width at 1500 HU. The images were reviewed at a designated post-processing station.

### 2.5. Pulmonary Severity Score

The extent of lung involvement was blindly evaluated by two expert radiologists (SP and AV) and classified according to the widely accepted score by Pan and coworkers [17]. CT findings were described using the Fleischner Society glossary and evaluated through a semiquantitative scoring system estimating pulmonary involvement. Each of the five lung lobes was visually scored on a scale of 0 to 5, with 0 indicating no involvement; 1, less than 5% involvement; 2, 5–25% involvement; 3, 26–49% involvement; 4, 50–75% involvement; and 5, more than 75% involvement. The final score was the result of the sum of each lobar score and could range from 0 to 25 (maximum involvement).

### 2.6. CAC CT Analysis

Coronary segments included the left main (LM) stem, the proximal, middle, and distal portions of the left anterior descending (LAD), left circumflex (LCX), and right coronary artery (RCA). The proximal LAD artery included the segment proximal to the first diagonal or the first centimeter of the artery if this branch was not visible. The middle LAD included the segment between the first and second diagonal, or the second centimeter of the artery if the diagonals were not visible. The distal LAD included the remaining part of the vessel. Similarly, the LCX artery was divided into proximal (up to first obtuse marginal or the first centimeter of the artery), middle (between first and second obtuse marginal or the second centimeter), and distal segment (the remaining vessel). Finally, the RCA was divided into proximal (horizontal), middle (vertical), and distal (horizontal component curving under the base of the heart, which, in some cases, included the posterior descending coronary artery). Radiologists and cardiologists independently performed segmented vessel-specific scoring by using an ordinal scale of 0–3 [18]. Inconsistencies were resolved by consensus. No CAC, mild, moderate, and heavy CAC were assigned scores of 0, 1, 2, and 3, respectively. CAC was classified as mild if there were only isolated flecks of calcium within a segment, severe if there was continuous calcium, and moderate if greater than what could be considered mild but less than the definition of severe. The ordinal values from all coronary segments were then summed to provide a total segmented vessel-specific score that ranged from 0 to 30: absence of CAC (score = 0), mild (score = 1–5), moderate (score = 6–11), and severe (score = 12–30).

### 2.7. Outcome Measures 

The primary outcome measure was the evaluation of moderate-to-severe CAC between cases and controls, selected according to the combination of 30-day all-cause mortality and the need for invasive ventilation during index hospitalization.

The secondary outcome measures were the analysis of moderate-to-severe CAC between groups, individually evaluating 30-day all-cause mortality and the need for invasive ventilation.

### 2.8. Sample Size Calculation and Statistics 

We estimated that the presence of moderate/severe CAC confers at least a 2.5-fold increase in the risk of death or need for invasive ventilation. To have a power of 80%, admitting an α error of 0.05, it was necessary to include 65 cases and 130 controls, for a final sample size of 195 patients [19].

Continuous variables were summarized as means and standard deviations for normally distributed variables, or as medians and interquartile ranges (IQR) for non-normally distributed variables, whereas categorical variables were presented as frequencies and corresponding percentages. Groupwise comparisons of continuous variables were performed using either the T-test and one-way analysis of variance, or the Wilcoxon rank sum test and the Kruskal Wallis test, for parametric and non-parametric data, respectively. Comparison between categorical variables was performed using the Pearson chi-square with continuity correction. Stepwise multiple logistic regression was used to assess the relationship between demographic and clinical, laboratory, and CT variables, as well as the primary endpoint. All variables with *p* < 0.1 on univariable analysis were considered for inclusion into multivariable logistic regression models. Prognostic accuracy of pulmonary severity score and CAC score were assessed by using AUC receiver operating characteristic (ROC) analysis to estimate sensitivity and specificity. ROC curve comparison was performed with the method of DeLong et al. [20]. The significance level was 5%. All statistical analyses were carried out using SPSS Statistics 26 (IBM, Armonk, NY, USA), and MedCalc version 22.026 (MedCalc Software Ltd., Ostend, Belgium).

## 3. Results

A total of 486 patients met the inclusion criteria. From this cohort, after 1:2 case-to-control matching, 195 patients were extracted and represented the final study population, the demographic and clinical data of which are shown in Table 1. Median age was 71.5 years, with 69 patients (35.4%) being female. In terms of cardiovascular risk factors, mean BMI was 25.1 kg/m^2^, 127 patients had arterial hypertension (65.1%), and 44 diabetes mellitus (16.5%). The median length of hospital stay was 13 days (IQR 7–22 days). The high-sensitivity troponin-T title was available in 66 out of 195 patients (33.8%), too few to analyze the influence of this marker on outcomes or even to apply an imputation procedure to replace the missing values.

### 3.1. Comparison between Cases and Controls

The 65 cases consisted of 43 patients dying within 30 days of being hospitalized and 22 patients undergoing mechanical ventilation. In 18 cases, death was preceded by the need for mechanical ventilation. The median length of hospital stay was 15 days (IQR 10–25). Controls were 130 age- and sex-matched patients who were alive 30 days after admission without requiring mechanical ventilation. The median length of stay for controls was 12 days (IQR 7–22). 

Table 1 presents the univariate comparison between groups. Baseline characteristics were similar, except for BMI, which was higher among cases. This group also had a higher respiratory rate, inflammation markers, a greater need for antibiotic therapy, and a lower PO2/FiO2 ratio. The median pulmonary severity score was significantly higher in the cases than the controls (10 vs. 8, respectively, *p* = 0.0001), as was the CAC score (5 vs. 2, *p* = 0.009).

**Figure 1:**
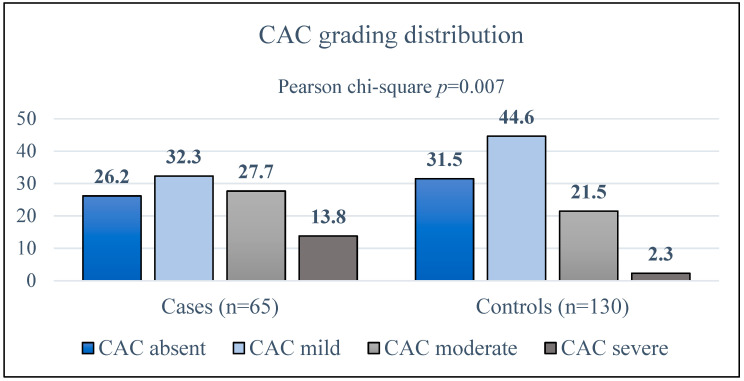
Coronary artery calcium (CAC) score distribution in cases and controls.

**Figure 2:**
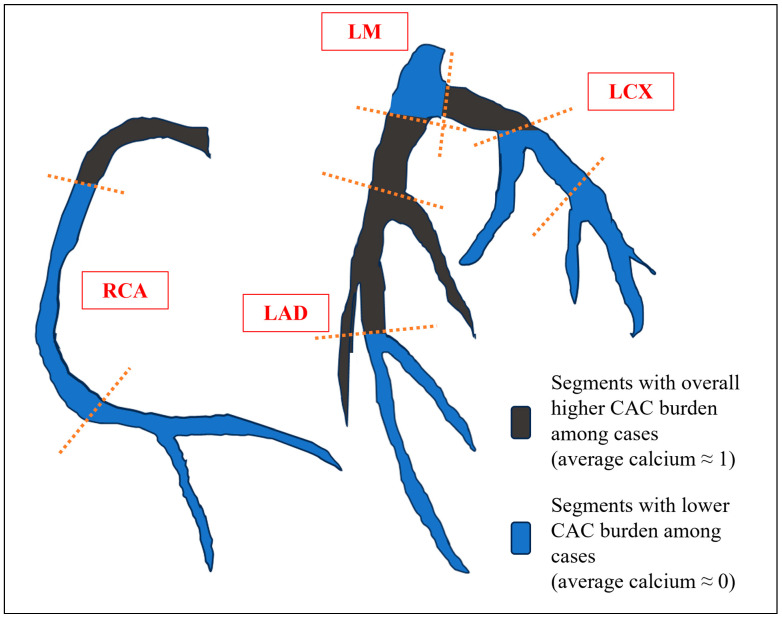
Anatomic recognition of the coronary segments with the highest calcium scores in the case group.

Figure 1 illustrates the distribution of CAC in the population. Notably, 41.5% of cases compared to 23.8% of controls had moderate-to-severe CAC (2-way Fisher’s exact test: *p* = 0.013). In the former group, the highest calcium burden was observed in the proximal epicardial coronary segments (Figure 2). 

### 3.2. Primary and Secondary Endpoint

**Figure 3:**
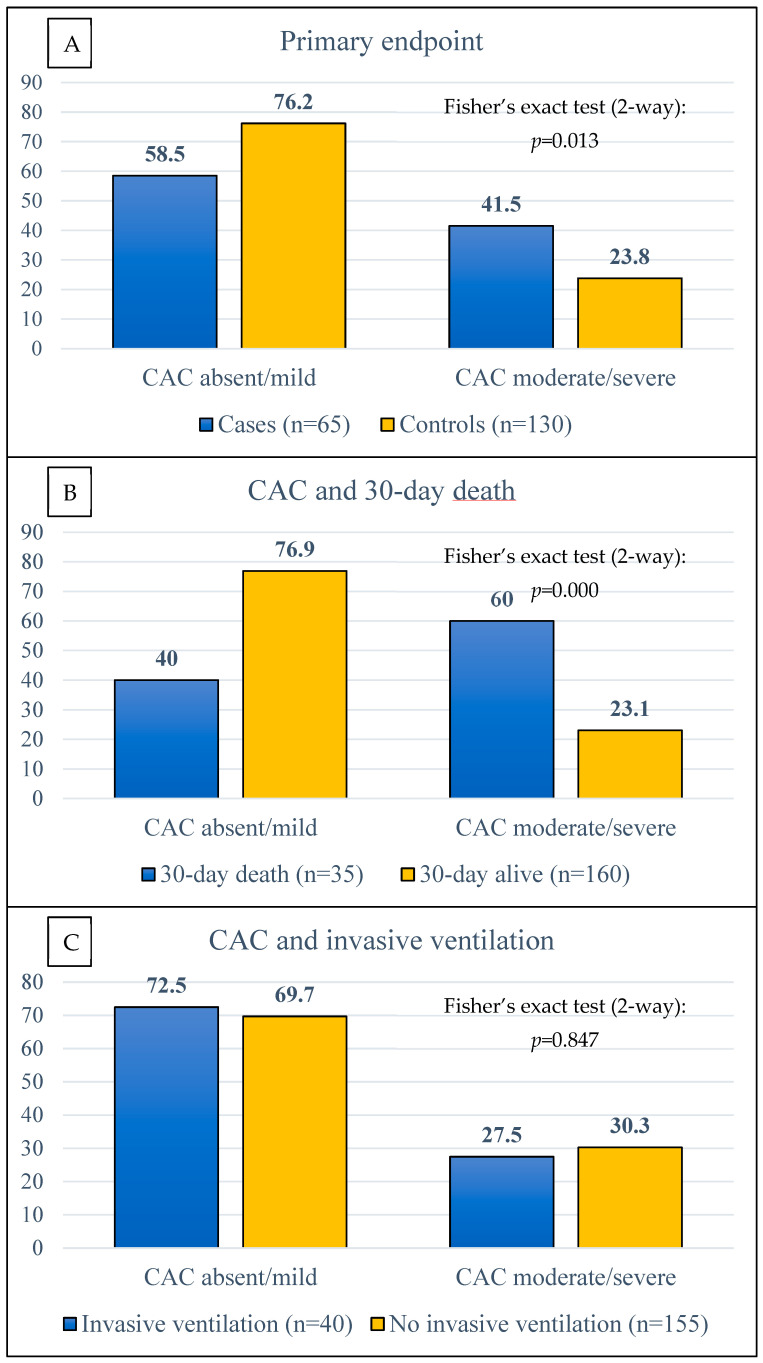
Primary endpoint of the study (**A**). Coronary artery calcium (CAC) distribution according to 30-day death (**B**) and need for invasive ventilation (**C**).

The prevalence of moderate-to-severe CAC was significantly higher in cases than in controls (41.5% vs. 23.8%; *p* = 0.013), as reported in Figure 3A. The analysis of the secondary endpoints (Figure 3B,C) demonstrates a similar rate of moderate-to-severe CAC between patients who received or did not receive invasive ventilation, but a higher prevalence in those who died within 30 days compared to survivors (60.0% vs. 23.1%; *p* = 0.000).

### 3.3. Primary Endpoint Predictors and Prognostic Accuracy

**Figure 4:**
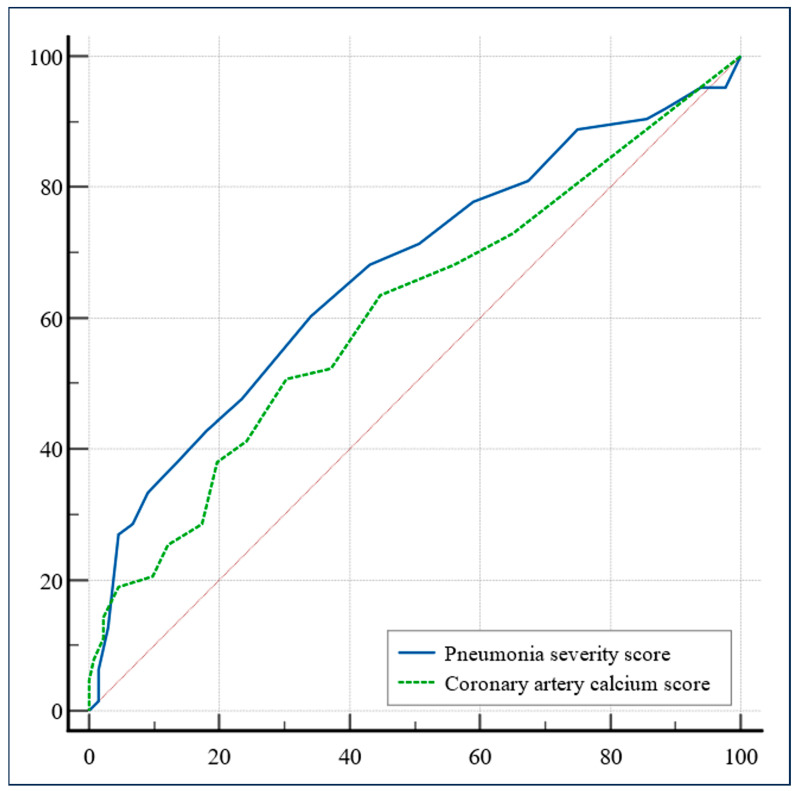
Receiver operating characteristic curve comparison between the two variables obtained from chest CT: pneumonia severity score and coronary artery calcium score.

White blood cell count, moderate-to-severe CAC, the need for antibiotic therapy, and severe pneumonia at CT scan were independent predictors of 30-day mortality and invasive ventilation (Table 2). ROC curve analysis showed that the area under the curve was similar: 0.668 for pulmonary severity score, and 0.609 for CAC score (difference between areas 0.596; 95% C.I.: −0.08 to 0.2; *p* = 0.388) (Figure 4).

## 4. Discussion

The chest CT scan is an essential tool for detecting interstitial pneumonia and assessing its severity, playing a crucial role throughout the COVID-19 pandemic. A careful analysis of CT scans obtained on admission in these often critically ill patients also provided interesting insights into the presence of CAC in this population. Some cohort studies have found a correlation between the presence and extent of CAC and patient outcomes in terms of all-cause mortality, need for mechanical ventilation, and ICU admission. However, other data have shown conflicting results [21]. The results of these studies have recently been summarized in a meta-analysis which included 3769 patients and showed an increased risk of mortality with a high CAC score compared to a low CAC score (RR, 2.74; 95% CI, 1.94–3.86; *p* < 0.00001) [22]. Therefore, it is unclear whether CAC severity, which may indicate the presence of obstructive coronary artery disease, is causally related to adverse outcomes or is merely a marker of poorer health status. This uncertainty was mainly due to the cohort design of these studies, which precluded the complete correction for the influences of unbalanced baseline variables. In fact, the extent and severity of CAC is age-dependent and higher in males than females. The present analysis was designed to at least partially overcome this limitation, being a case-control study with age- and sex-matching. We found that CAC was an independent predictor of death and the need for invasive mechanical ventilation in patients with COVID-19-related interstitial pneumonia, along with pulmonary CT score severity, white blood cell (WBC) count, and the need for antibiotic therapy. The last three variables have a pathophysiological connection based on the pivotal role of the inflammatory response during the SARS-CoV-2 infection [23]. Patients with enhanced dysregulated inflammation are arguably those with greater pulmonary tissue involvement, higher WBC counts, and who require antibiotic treatment due to superimposed bacterial infections [24]. The significant predictive role of CAC is confirmed in this case-control study, consistent with findings from other retrospective cohort studies [8,10,11,12]. The usefulness of CAC quantification in COVID-19 patients has several implications. First, it can expedite the patient diagnostic work-out, with the possibility of identifying those at risk for cardiac complications. In fact, given that CAC is an indicator of underlying atherosclerotic coronary artery disease, which is strictly correlated with the prevalence of cardiovascular risk factors like diabetes mellitus and hypertension, patients with a higher calcium burden may develop myocardial injury, cardiac dysfunction, and arrhythmias [25]. As a consequence, this stratification tool can help to establish whether patients should be admitted to a standard medical ward or intensive care unit. Second, patient stratification can also help identify those with an indication to start specific antiviral and corticosteroid therapy soon after hospital admission [26,27]. On the other hand, as CAC is a marker of coronary atherosclerosis and is associated with an increased risk of myocardial injury during severe COVID-19, these patients may also face higher risks of myocardial toxicity and complications, such as arrhythmias and death, when undergoing hydroxychloroquine therapy [28]. 

It is worth noting that, unlike other studies [29], our analysis accounted for the relative influence of the severity of interstitial pneumonia, expressed as lung involvement at CT scan, which was included in the multivariate model and emerged as an independent predictor of outcome, similar to CAC. Interestingly, the ROC curve comparison did not find a significant difference between the scores of CAC severity and pulmonary severity, a known independent predictor of outcome [30], strengthening the stratification role of CAC. Finally, CAC was a predictor of death, but not of the need for invasive ventilation. Only in 61.5% of cases (40 out of 65 patients) was death preceded by the need for invasive ventilation, possibly indicating that the cause of death might be cardiac related rather than respiratory in the remaining population.

The two age- and sex-matched groups differed primarily in baseline characteristics, showing a higher inflammatory status in the cases, in addition to higher body mass index and more frequent history of cancer. Some studies found an association between CAC, which reflects the risk of coronary atherosclerosis, and certain cancer subtypes such as lung and colorectal cancer [31]. A similar association was found with obesity [32]. This is not surprising, as these diseases share common risk factors such as smoking, sedentary lifestyles, diets high in saturated fats, and chronic inflammation. However, our multivariate analysis did not show obesity and cancer history as independent predictors of a poor 30-day outcome.

We focused on the first wave of COVID-19, which featured more severe infection and a higher number of adverse events. Indeed, throughout the pandemic, most countries applied measures to limit the spread of the virus, including social distancing, temporary interruption of entire work and leisure sectors, and distribution of personal protective equipment, while awaiting commercialization of specific vaccines and treatments. In the meantime, the appearance of several viral variants with lower lethality rates was observed. Our findings may be applicable to different pulmonary diseases that share a common inflammatory substrate and result in interstitial pneumonia. In acute contexts, e.g., Middle East respiratory syndrome (MERS) (caused by MERS-CoV) and influenza, infections typically severely affect patients with hypertension, diabetes, and cardiovascular disease. Moreover, these diseases share similar mechanisms leading to myocardial damage as well as the potential risk of dysregulated inflammatory response [3]. In chronic contexts, some noninfectious etiologies could also fit within the same perspective. Wang et al. reported that patients with interstitial lung disease associated with inflammatory connective tissue disorders had higher cardiovascular involvement, inflammation markers, and prolonged disease [33]. Bray and colleagues, analyzing the high-resolution chest CT scans of patients suffering from idiopathic pulmonary fibrosis, found a higher coronary calcium burden compared to non-smokers, a greater risk of coronary artery disease (CAD), and suggested considering CAD screening for the potential prevention of cardiac events [34]. The potential application of CAC CT analysis to study patients with interstitial pneumonia, irrespective of the underlying etiology, supports the renewed interest in this area of research.

The topic of risk stratification may include other patient characteristics, including peripheral arterial disease, which shares physiopathological and prognostic similarities with coronary artery disease. The presence of calcium in peripheral vascular districts, its correlation with CAC, and its potential impact on the outcomes of COVID-19 patients may be a field of study in the near future [35,36,37].

## 5. Limitations

Severe coronary artery disease documented by CAC in COVID-19 patients may be responsible for acute cardiac events caused by an oxygen demand/supply imbalance. However, other cardiac complications may influence patient outcomes, e.g., coronary thrombosis, myocarditis, and Takotsubo syndrome. All these conditions have a severe prognosis and imply intensified treatment [3]. In addition to CT scans, other diagnostic investigations may be required, including echocardiography, cardiac magnetic resonance imaging, or invasive coronary angiography, depending on the clinical presentation. Nevertheless, semi-quantitative CAC assessment obtained by chest CT analysis is valuable for initial risk stratification and differential diagnosis, since such information can be obtained with standard and globally available equipment [38]. 

The case-control design sought to reduce the age-related bias compared to cohort studies. However, despite CAC being associated with a worse outcome in our study, in line with previous observational ones, we cannot estimate the CAC prognostic effect across different ages. The mean age in our cohort was 71 years, but in a large study with a mean age of 63, CAC still demonstrated a correlation with outcomes [29]. We can assume that CAC is associated with a worse prognosis even in younger patients, but the power of this association might be different.

We acknowledge that assessing CAC severity (similarly to the severity of pulmonary involvement) cannot be considered in isolation for accurate prognostic stratification, given the low value of the C-index, which indicates the need for an integrated approach to risk evaluation.

Finally, in our study, CAC severity was assessed using a semi-quantitative technique rather than an objective, quantitative method like the Agatston score. However, the former was validated in several studies, and the two different approaches are highly correlated and reproducible [18,39].

## 6. Conclusions

This case-control study demonstrated that the CAC burden was higher in COVID-19 patients who did not survive 30 days or who required mechanical ventilation, with CAC playing an independent prognostic role comparable to the pulmonary severity score.

## Figures and Tables

**Table 1:** Univariate comparison between cases and controls.

Clinical Characteristics	Total (N = 195)	Cases (n = 65)	Controls (n = 130)	*p*-Value
Age, years	71.5	71.6	71.4	0.93
Female (n, %)	69 (35.4%)	23 (35.4%)	46 (35.4%)	1.00
BMI (kg/m^2^) *	25.1 (±5.0)	26.6 (±6.5)	24.4 (±3.8)	0.013
Hypertension (n, %)	127 (65.1%)	47 (72.3%)	80 (61.5%)	0.15
Diabetes (n, %)	44 (22.5%)	15 (23.1%)	29 (22.3%)	0.46
Smoker (n, %)	64 (32.8%)	21 (32.3%)	43 (33.1%)	0.91
COPD (n, %)	22 (11.3%)	8 (12.3%)	14 (10.8%)	0.749
CCS (n, %)	26 (13.3%)	7 (10.8%)	19 (14.6%)	0.602
Cancer (n, %)	26 (13.3%)	14 (21.5%)	12 (9.2%)	0.017
ACEI/ARB use (n, %)	81 (41.5%)	23 (35.4%)	58 (44.6%)	0.22
Respiratory rate > 24/min (n, %)	59 (30.3%)	32 (49.2%)	27 (20.8%)	0.0001
PO2/FiO2 ratio *	321 (±117)	278 (±125)	334 (±102)	0.027
White blood cells, ×10^3^/µL	6.7 (5.0–9.1)	8.5 (6.1–10.8)	6.1 (4.6–7.8)	0.0001
Platelets, ×10^3^/µL	210 (189–256)	21 (172–258)	209 (167–264)	0.748
Lymphocytes, ×10^3^/µL	0.97 (0.67–1.4)	0.94 (0.60–1.62)	1.0 (0.68–1.40)	0.463
Creatinine, mg/dL	0.98 (0.79–1.20)	0.93 (0.60–1.63)	0.93 (0.77–1.14)	0.035
D-dimer, µg/L	926 (500–2540)	1804 (739–3554)	773 (434–1974)	0.001
CRP, mg/L	56.3 (22.5–103)	79.7 (38.5–155)	51.1 (13.5–82.4)	0.001
Ferritin, µg/L	567 (159–1119)	863 (888–1645)	314 (66–895)	0.0001
LDH, IU/L	287 (227–392)	369 (278–450)	260 (212–362)	0.001
Admission hs-Troponin T, ng/L	18.0 (10.0–46.5)	21.5 (12.0–62.5)	17.0 (10.0–32.0)	0.233
Antibiotic therapy (n, %)	110 (56.4%)	50 (76.9%)	60 (42.6%)	0.0001
Antiviral therapy (n, %)	109 (55.9%)	39 (60%)	70 (53.8%)	0.415
Hydroxychloroquine (n, %)	132 (66.7%)	48 (73.8%)	84 (64.6%)	0.194
Corticosteroid (n, %)	93 (47.7%)	41 (63.1%)	52 (40.0%)	0.002
Tocilizumab (n, %)	49 (25.1%)	20 (30.8%)	29 (22.3%)	0.199
Pulmonary severity score	9 (5–12)	10 (7–16)	8 (4–10)	0.0001
CAC score	3 (0–7)	5 (0–8.5)	2 (0–5)	0.009

Legend: BMI: body mass index; COPD = chronic obstructive pulmonary disease; CCS = chronic coronary syndrome; C-RP = C-reactive protein; LDH = lactate dehydrogenase; hs = high sensitivity; CAC = coronary artery calcium. Continuous variables are reported as median and interquartile range, except for * as mean ± standard deviation.

**Table 2:** Independent primary endpoint predictors.

30-Day Death and Need for Invasive Ventilation		
	OR (95% CI)	*p*
White blood cell count	3.33 (1.34–8.30)	0.010 *
Creatinine	2.21 (0.93–5.20)	0.070
D-dimer	2.14 (0.84–5.47)	0.111
C-reactive protein	2.36 (0.99–5.69)	0.055
Ferritin	0.76 (0.27–2.15)	0.603
Lactate dehydrogenase	1.66 (0.62–4.44)	0.312
Cancer history	1.94 (0.84–4.11)	0.155
Body mass index	1.62 (0.70–3.81)	0.266
Antibiotic therapy	3.30 (1.33–8.22)	0.010 *
Corticosteroid	1.82 (0.74–4.49)	0.193
PO2/FiO2 ratio	0.73 (0.29–1.86)	0.513
Respiratory rate > 24/min	0.36 (0.00–1.46)	0.79
Moderate-to-severe CAC	2.55 (1.01–6.46)	0.048 *
Severe pneumonia at CT scan	4.02 (1.41–11.41)	0.009 *

CAC: coronary artery calcium; CT: computed tomography; OR: odds ratio; * statistically significant.

## Data Availability

Anonymized data will be made available by the corresponding author upon reasonable request after publication.
